# Order isomophisms between Riesz spaces

**DOI:** 10.1007/s11117-018-0560-y

**Published:** 2018-01-27

**Authors:** B. L. van Engelen, A. C. M. van Rooij

**Affiliations:** 1 Zijpendaalseweg 25, 6814 CC Arnhem, The Netherlands; 2 Department of Mathematics, Radboud University Nijmegen, P.O. Box 9010, 6500 GL Nijmegen, The Netherlands

**Keywords:** Riesz spaces, Order isomorphisms, Universal completion, 06-02

## Abstract

The first aim of this paper is to give a description of the (not necessarily linear) order isomorphisms $C(X)\rightarrow C(Y)$ where *X*, *Y* are compact Hausdorff spaces. For a simple case, suppose *X* is metrizable and *T* is such an order isomorphism. By a theorem of Kaplansky, *T* induces a homeomorphism $\tau :X\rightarrow Y$. We prove the existence of a homeomorphism $X\times \mathbb {R}\rightarrow Y\times \mathbb {R}$ that maps the graph of any $f\in C(X)$ onto the graph of *Tf*. For nonmetrizable spaces the result is similar, although slightly more complicated. Secondly, we let *X* and *Y* be compact and extremally disconnected. The theory of the first part extends directly to order isomorphisms $C^{\infty }(X)\rightarrow C^{\infty }(Y)$. (Here $C^{\infty }(X)$ is the space of all continuous functions $X\rightarrow [-\infty ,\infty ]$ that are finite on a dense set.) The third part of the paper considers order isomorphisms *T* between arbitrary Archimedean Riesz spaces *E* and *F*. We prove that such a *T* extends uniquely to an order isomorphism between their universal completions. (In the absence of linearity this is not obvious.) It follows, that there exist an extremally disconnected compact Hausdorff space *X*, Riesz isomorphisms $\hat{}$ of *E* and *F* onto order dense Riesz subspaces of $C^{\infty }(X)$ and an order isomorphism $S:C^{\infty }(X)\rightarrow C^{\infty }(X)$ such that $\hat{Tf}=S\hat{f}$ ($f\in E$).

## Introduction

Two Riesz spaces may be order isomorphic without being Riesz isomorphic, an example being formed by $l^1$ and $l^2$. However, order isomorphic Riesz spaces necessarily share certain properties that at first sight seem to depend on the Riesz space structure and not only on the ordering. For instance, if two Archimedean Riesz spaces are order isomorphic, then their universal completions (in the sense of [1]) are order and even Riesz isomorphic, although the definition of the universal completion involves the vector space structure.

In this paper we consider order isomorphisms between Riesz spaces. We start with the Riesz spaces *C*(*X*), *C*(*Y*) where *X* and *Y* are compact Hausdorff spaces. A homeomorphism $\pi :Y\rightarrow X$ induces a linear order isomorphism $f\rightarrowtail f\circ \pi$ of *C*(*X*) onto *C*(*Y*). Conversely, I. Kaplansky proves in [2] that *X* and *Y* are homeomorphic as soon as there exists an order isomorphism $C(X)\rightarrow C(Y)$, linear or not. In Sect. 2, we investigate the order isomorphisms $C(X)\rightarrow C(Y)$.

As a (not quite representative) case in point, consider $X=Y=[0,1]$. We will see that an order isomorphism $T:C([0,1])\rightarrow C([0,1])$ engenders an $F:[0,1]\times \mathbb {R}\rightarrow [0,1]\times \mathbb {R}$ that maps the graph of any $f\in C([0,1])$ onto the graph of *Tf*. We say that *T* “acts horizontally” (or “vertically”) if for every point of $[0,1]\times \mathbb {R}$ we obtain its image under *F* by shifting horizontally (vertically). For instance, the formulasdescribe order isomorphisms $C([0,1])\rightarrow C([0,1])$; *S* acts horizontally, *R* vertically. (The maps *F* corresponding to *S* and *R* are the inverses of $(x,y)\rightarrowtail (x^2,y)$ and $(x,y)\rightarrowtail (x,e^{x}y^3)$, respectively.) It turns out that every order isomorphism $C([0,1])\rightarrow C([0,1])$ is uniquely a product $T_{2}\circ T_{1}$ where $T_{1}$ acts horizontally, $T_{2}$ acts vertically.

For *X* and *Y* distinct from [0, 1] the sailing may be less smooth. In general, an order isomorphism $C(X)\rightarrow C(Y)$ can be described in terms of a homeomorphism $X_{0}\times \mathbb {R} \rightarrow Y_{0}\times \mathbb {R}$ where $X_{0}$ and $Y_{0}$ are obtained from *X* and *Y* by removing certain meagre sets.

In Sect. 3, *X* and *Y* are extremally disconnected and we prove analogues of the above results for order isomorphisms $C^{\infty }(X)\rightarrow C^{\infty }(Y)$. The purpose of that lies in Sect. 4, where we look at an order isomorphism *T* between arbitrary Archimedean Riesz spaces *E* and *F*. We show that such a *T* extends uniquely to an order isomorphism between the universal completions of *E* and *F*. These universal completions are Riesz isomorphic to some $C^{\infty }(X)$ and we can apply the results of Sect. 3.

For $s\in \mathbb {R}$ we denote by $\overline{s}$ the constant function with value *s* (on any given set).

## Kaplansky’s theorem

### Definition 1

Let *X* and *Y* be compact Hausdorff spaces. A homeomorphism $\pi :Y\rightarrow X$ induces an order isomorphism $\pi ^*:C(X)\rightarrow C(Y)$:

$$\begin{aligned} \pi ^*f=f\circ \pi \quad \quad (f\in C(X)). \end{aligned}$$

In the reverse direction we have:

### Theorem 2

(Kaplansky [2]) Let *X* and *Y* be compact Hausdorff spaces such that *C*(*X*) and *C*(*Y*) are order isomorphic. Then *X* and *Y* are homeomorphic.

### Proof

(Our proof is Kaplansky’s in disguise but it yields some extra information)

1. Let $a\in X$, $g\in C(X)$ and $T:C(X)\rightarrow C(Y)$ be an isomorphism. Claim: There exists a $b\in Y$ with
  $$\begin{aligned} f\in C(X), f(a)<g(a) \quad \Rightarrow \quad (Tf)(b)\le (Tg)(b). \end{aligned}$$
  Indeed, suppose no such *b* exists. For every $b\in Y$, we choose $f_{b}\in C(X)$ with $f_{b}(a)<g(a)$, $(Tf_{b})(b)>(Tg)(b)$. By compactness, there exist $b_{1},...,b_{N}$ in *Y* such that $Y=\bigcup _{1\le n\le N}\{y:(Tf_{b_{n}})(y)>(Tg)(y)\}$. Putting $f:=f_{b_{1}}\vee ...\vee f_{b_{N}}$ we get $f(a)<g(a)$ but $Tf\ge Tg$. The latter inequality implies that $f\ge g$: a contradiction.
2. Let $a\in X$, $g,h\in C(X)$, $b,c\in Y$, and assume:
  $$\begin{aligned} \begin{aligned} f\in C(X), f(a)<g(a) \quad \Rightarrow \quad (Tf)(b)\le (Tg)(b), \\ f\in C(X), f(a)<h(a) \quad \Rightarrow \quad (Tf)(c)\le (Th)(c). \end{aligned} \end{aligned}$$
  Claim: $b=c$. For a proof, suppose $b\not = c$. Choose any $f\in C(X)$ with $f(a)<g(a)\wedge h(a)$. There exist $j,k \in C(Y)$ such that $j(b)>(Tg)(b)$, $k(c)>(Th)(c)$, $j\wedge k = Tf$. Then $(T^{-1}j)(a)\ge g(a)$, $(T^{-1}k)(a)\ge h(a)$, $(T^{-1}j)\wedge (T^{-1}k)=f$. But that contradicts $f(a)<g(a)\wedge h(a)$.
3. It follows from (1) and (2) that, for a given $a\in X$, there is a unique $b\in Y$ with
  $$\begin{aligned} f,g\in C(X), f(a)<g(a) \quad \Rightarrow \quad (Tf)(b)\le (Tg)(b). \end{aligned}$$
4. This enables us to define a map $\sigma :X\rightarrow Y$ by:
  $$\begin{aligned} x\in X, f,g\in C(X), f(x)<g(x) \quad \Rightarrow \quad (Tf)(\sigma x)\le (Tg)(\sigma x). \end{aligned}$$
  Similarly, there is a $\tau :Y\rightarrow X$, determined by:
  $$\begin{aligned} y\in Y, f,g\in C(Y), f(y)<g(y) \quad \Rightarrow \quad (T^{-1}f)(\tau y)\le (T^{-1}g)(\tau y). \end{aligned}$$
5. Let $A\subseteq X$ and let *b* lie in the closure of $\sigma (A)$ in *Y*; we prove that $\tau b$ lies in the closure of *A*. To this end, choose $j_{1},j_{2}$ in *C*(*X*) with $(Tj_{1})(b)<(Tj_{2})(b)$. For all $f\in C(X)$ we have
  $$\begin{aligned} \begin{aligned}&f(x)<j_{1}(x) \quad \quad (x\in A) \\&\quad \Rightarrow \quad (Tf)(y)\le (Tj_{1})(y) \quad \quad (y\in \sigma (A)) \\&\quad \Rightarrow \quad (Tf)(b)\le (Tj_{1})(b)<(Tj_{2})(b) \\&\quad \Rightarrow \quad f(\tau b)\le j_{2}(\tau b). \end{aligned} \end{aligned}$$
  It follows that $\tau b$ lies in the closure of *A*.
6. For $a\in X$, by taking $A:=\{a\}$ and $b:=\sigma a$ one obtains $\tau \sigma a =a$. Thus $\tau \circ \sigma$ is the identity map of *X*. Similarly $\sigma \circ \tau$ is the identity map of *Y*. Letting *A* be an arbitrary closed subset of *X* and observing that $\sigma =\tau ^{-1}$ one sees that $\tau$ is continuous. So is $\sigma$. $\square$

### Observation 3

In (3) of the above proof it is shown that for every $x\in X$, there is precisely one $y\in Y$ with

$$\begin{aligned} f,g\in C(X), f(x)<g(x)\quad \Rightarrow \quad (Tf)(y)\le (Tg)(y). \end{aligned}$$

### Definition 4

Let *X* and *Y* be compact Hausdorff spaces. A homeomorphism $\sigma :X\rightarrow Y$ is said to be *associated* with an order isomorphism $T:C(X)\rightarrow C(Y)$ if:

$$\begin{aligned} x\in X, f,g\in C(X), f(x)<g(x) \quad \Rightarrow \quad (Tf)(\sigma x)\le (Tg)(\sigma x). \end{aligned}$$

By the above proof of Theorem 2, for every order isomorphism $C(X)\rightarrow C(Y)$ there is exactly one homeomorphism $X\rightarrow Y$ associated with it.

If $\sigma :X\rightarrow Y$ is associated with an order isomorphism *T*, then $\sigma ^{-1}$ is associated with $T^{-1}$.

Furthermore, if $\phi$ is a homeomorphism $Y\rightarrow X$, then the homeomorphism associated with $\phi ^*$ is $\phi ^{-1}$.

In the remainder of this section *X* and *Y* are compact Hausdorff spaces, *T* is an order isomorphism $C(X)\rightarrow C(Y)$, and $\sigma$ is the associated homeomorphism $X\rightarrow Y$.

### Lemma 5

Let $s,t\in \mathbb {R}$, $s<t$. Then the closed set $A:=\{y\in Y:(T\overline{s})(y)=(T\overline{t})(y)\}$ has empty interior.

### Proof

1. Assume $s=0$, $T\overline{0}=\overline{0}$. Then $A=\{y\in Y:(T\overline{t})(y)=0\}$. Suppose the interior of *A* is non-empty. Choose a non-zero *f* in $C(Y)^+$ that vanishes everywhere outside *A*. Then $f\wedge T\overline{t}=\overline{0}$, so $T^{-1}f\wedge \overline{t}=\overline{0}$. It follows that $T^{-1}f=\overline{0}$, so $f=\overline{0}$. Contradiction.
2. For the general case, put $t':=t-s$ and let $T'$ be the order isomorphism $f\rightarrowtail T(f+\overline{s})-T\overline{s}$ of *C*(*X*) onto *C*(*Y*), with $T'(\overline{0})=\overline{0}$. Then $T'\overline{t'}=T\overline{t}-T\overline{s}$, so $A=\{y\in Y:(T'\overline{t'})(y)=0\}$. Now by the first part, *A* has empty interior. $\square$

### Definition 6

Let $\sigma :X\rightarrow Y$ be the homeomorphism associated with *T*. Define the subset $X_{0}$ of *X* by

$$\begin{aligned} X\backslash X_{0}:=\bigcup _{\begin{array}{c} s,t\in \mathbb {Q} \\ s<t \end{array}} \{x:(T\overline{s})(\sigma x)=(T\overline{t})(\sigma x)\}\cup \bigcup _{\begin{array}{c} s,t\in \mathbb {Q} \\ s<t \end{array}} \{x:(T^{-1}\overline{s})(x)=(T^{-1}\overline{t})(x)\}. \end{aligned}$$

It follows from Lemma 5 that $X\backslash X_{0}$ is meagre (= first category). In particular, $X_{0}$ is dense in *X*.

### Lemma 7

Let $X_{0}$ be as above. Let $x\in X_{0}$, $f,g\in C(X)$. Then

1. $$f(x)<g(x) \Leftrightarrow (Tf)(\sigma x)<(Tg)(\sigma x)$$.
2. $$f(x)=g(x) \Leftrightarrow (Tf)(\sigma x)=(Tg)(\sigma x)$$.

### Proof

Suppose $f(x)<g(x)$. Choose $s,t\in \mathbb {Q}$ with $f(x)<s<t<g(x)$. By Definition 4, $(Tf)(\sigma x)\le (T\overline{s})(\sigma x)$ and $(T\overline{t})(\sigma x)\le (Tg)(\sigma x)$. As $x\in X_{0}$, $(T\overline{s})(\sigma x)<(T\overline{t})(\sigma x)$. Hence $(Tf)(\sigma x)<(Tg)(\sigma x)$. The proof of the reverse implication is similar.

The second part of the lemma follows. $\square$

### Corollary 8

Let $U\subseteq X$ be open. Let $f,g\in C(X)$.

1. $$f\le g$$ on $$U \Leftrightarrow Tf\le Tg$$ on $$\sigma (U)$$.
2. $$f=g$$ on $$U \Leftrightarrow Tf=Tg$$ on $$\sigma (U)$$.

### Proof

Suppose $f\le g$ on *U*. By Lemma 7, the open set $\{x\in U: (Tf)(\sigma x)>(Tg)(\sigma x)\}$ does not intersect the dense set $X_{0}$ and therefore must be empty, i.e., $Tf\le Tg$ on $\sigma (U)$.

The rest of the corollary follows. $\square$

### Notation 9

Let *Z* be a compact Hausdorff space. For $f\in C(Z)$ we let ${\text {supp}}(f)$, the support of *f*, be the closure of the set $\{z\in Z:f(z)\not = 0\}$.

For open $U\subseteq Z$ we put

$$\begin{aligned} B_{U}:= \{f\in C(Z):f=\overline{0} \text { on } U\}. \end{aligned}$$

$B_{U}$ is a band of the Riesz space *C*(*Z*). Every band of *C*(*Z*) is $B_{U}$ for some open $U\subseteq Z$ (see [3], Exercise 22.10).

If $f\in C(Z)$ and $U\subseteq Z$ is open, then

$$\begin{aligned} f\in B_{U} \Leftrightarrow {\text {supp}}(f)\subseteq Z\backslash U. \end{aligned}$$

### Corollary 10

Assume $T\overline{0}=\overline{0}$ and let $\sigma :X\rightarrow Y$ be associated with *T*.

1. For every $f\in C(X)$, $\sigma$ maps ${\text {supp}}(f)$ onto ${\text {supp}}(Tf)$.
2. If $U\subseteq X$ is open, then $T(B_{U})=B_{\sigma {U}}$.

### Proof

1. Take $X_{0}$ as above. Let *W* be $\{x\in X:f(x)\not = 0\}$. By Lemma 7 and by the assumption that $T\overline{0}=\overline{0}$, $\sigma$ maps $W\cap X_{0}$ into $\{y\in Y:(Tf)(y)\not = 0\}$, so $W\cap X_{0}\subseteq \sigma ^{-1}({\text {supp}}(Tf))$. But $X_{0}$ is dense in *X* and *W* is open. Then the closure of $W\cap X_{0}$ is the same as the closure of *W*, which is ${\text {supp}}(f)$. Then ${\text {supp}}(f)\subseteq \sigma ^{-1}({\text {supp}}(Tf))$ and thus $\sigma ({\text {supp}}(f))\subseteq {\text {supp}}(Tf)$. As $\sigma ^{-1}$ is the homeomorphism associated with $T^{-1}$ (see Definition 4) we also have $\sigma ^{-1}({\text {supp}}(Tf))\subseteq {\text {supp}}(f)$ and we are done.
2. Follows from (1). $$\square $$

### Theorem 11

(Functoriality) Suppose *Z* is a third compact Hausdorff space and *S* is an order isomorphism $C(Y)\rightarrow C(Z)$. Let $\sigma :X\rightarrow Y$ and $\omega :Y\rightarrow Z$ be the homeomorphisms associated with *T* and *S*, respectively. Then $\omega \circ \sigma$ is the homeomorphism $X\rightarrow Z$ associated with *ST*.

### Proof

Let $\phi :X\rightarrow Z$ be the homeomorphism associated with *ST*. Form $X_{0}$ as in Definition 6. Let $x\in X_{0}$. If $f,g\in C(X)$ and $f(x)<g(x)$, then $(Tf)(\sigma x)<(Tg)(\sigma x)$ by Lemma 7, so that $(STf)(\omega \sigma x)\le (STg)(\omega \sigma x)$. Thus $\omega \sigma x=\phi x$.

We see that $\omega \circ \sigma$ and $\phi$ coincide on the dense subset $X_{0}$ of *X*. Then $\omega \circ \sigma =\phi$. $\square$

### Definition 12

We say that *T* acts *horizontally* if $T\overline{s}=\overline{s}$ for all $s\in \mathbb {R}$ and (in case $X=Y$) that *T* acts *vertically* if the homeomorphism associated with *T* is the identity map of *X*.

If $\sigma :X\rightarrow Y$ is a homeomorphism, then the isomorphism $\sigma ^*$ (see Definition 4) acts horizontally. If $X=Y$ and $u\in C(X)$, then the translation map $f\rightarrowtail f+u$ is an order isomorphism $C(X)\rightarrow C(X)$ that acts vertically.

### Comment 13

1. Kaplansky’s original proof of Theorem 2 is quite roundabout. The proof we give yields a more direct connection between the order isomorphism *T* and the homeomorphism $\tau$. Our Corollary 8(2) is essentially the characterisation of $\tau$ obtained by Geuze in his thesis [4].
2. Kaplanksy’s Theorem is more general than our Theorem 2, considering spaces of continuous functions with values in an arbitrary chain instead of $\mathbb {R}$ (in which case the spaces *X* and *Y* have to satisfy natural regularity conditions). See also [4] for further generalisations.

## Vertically acting order isomorphisms

In this section, *X*, *Y*, *T* are as above.

### Lemma 14

Let $\sigma :X\rightarrow Y$ be the homeomorphism associated with *T*. Then the isomorphisms $T\circ \sigma ^*:C(Y)\rightarrow C(Y)$ and $\sigma ^* \circ T:C(X)\rightarrow C(X)$ are acting vertically.

### Proof

Apply Theorem 11 together with the fact that $\sigma ^{-1}$ is associated with $\sigma ^*$ (see Definition 4). $\square$

### Corollary 15

If *T* acts horizontally and $\sigma$ is the associated homeomorphism, then $T=(\sigma ^{-1})^*$.

### Proof

Let $f\in C(X), x\in X$. For $s\in \mathbb {R}$ with $s>f(x)$ we have $f(x)<\overline{s}(x)$. Consequently, $(Tf)(\sigma x)\le (T\overline{s})(\sigma x) =\overline{s}(\sigma x)=s$. It follows that $(Tf)(\sigma x)\le f(x)$. Similarly, $(Tf)(\sigma x)\ge f(x)$. Therefore, $(Tf)(\sigma x)=f(x)$.

Consequently, $$Tf=f\circ \sigma ^{-1}$$ $$(f\in C(X))$$, i.e., $$T=(\sigma ^{-1})^*$$. $$\square $$

### Corollary 16

(for $X=Y$) The only order isomorphism $C(X)\rightarrow C(X)$ that acts both horizontally and vertically is the identity map.

### Theorem 17

Let $X=Y$, $T\overline{0}=\overline{0}$. Then the following are equivalent:

1. *T* acts vertically.
2. *T* is support preserving i.e. ${\text {supp}}(Tf)={\text {supp}}(f) \quad (f\in C(X))$.
3. *T* maps every band of *C*(*X*) onto itself.

### Proof

$(1)\Rightarrow (2)$ is a special case of Corollary 10.

$(2)\Rightarrow (3)$ Let *B* be a band. There is an open set *U* with $B=B_{U}$. For $f\in C(X)$ we then have $f\in B \Leftrightarrow f\in B_{U} \Leftrightarrow {\text {supp}}(f)\subseteq X\backslash U \Leftrightarrow {\text {supp}}(Tf)\subseteq X\backslash U \Leftrightarrow Tf\in B$. Thus, $T(B)=B$.

$(3)\Rightarrow (1)$ Let $\sigma$ be the homeomorphism associated with *T*. Suppose there is an $x\in X$ with $\sigma x\not =x$. Choose $f\in C(X)$ so that $f=\overline{0}$ on an open set *U* containing *x*, and $f=\overline{1}$ on an open set *V* containing $\sigma x$. Then $f\in B_{U}$, so (by (3) and by Corollary 10(2)) $f\in T(B_{U})=B_{\sigma (U)}$. But $\sigma x\in \sigma (U)$ and $f(\sigma x)=1$. Contradiction. $\square$

We proceed to prove the representation theorem announced in the Introduction.

### Theorem 18

Let $X=Y$ and let *T* be acting vertically. Take $X_{0}$ as in Definition 6. Define $F:X_{0}\times \mathbb {R}\rightarrow X_{0}\times \mathbb {R}$ by

$$\begin{aligned} F(x,s):=(x,(T\overline{s})(x)) \quad \quad (x\in X_{0},s\in \mathbb {R}). \end{aligned}$$

Then *F* is a homeomorphism and for every $f\in C(X)$ we have

1 $$\begin{aligned} (x,(Tf)(x))=F(x,f(x)) \quad \quad (x\in X_{0}). \end{aligned}$$

Putting it differently: if for $g\in C(X)$ we define $\Gamma _{g}:=\{(x,y)\in X_{0}\times \mathbb {R}: y=g(x)\}$ (so that $\Gamma _{g}$ is dense in the graph of *g*), then *F* maps $\Gamma _{f}$ onto $\Gamma _{Tf}$ ($f\in C(X)$).

### Proof

1. If $x\in X_{0}$, $f\in C(X)$ and $s=f(x)$, then $(Tf)(x)=(T\overline{s})(x)$ by Lemma 7. This proves Eq. (1).
2. Take $x\in X_{0}$ and consider the function $\phi :s\rightarrowtail (T\overline{s})(x)$ $(s\in \mathbb {R})$. It follows from Lemma 7 that $\phi$ is strictly increasing. For $t\in \mathbb {R}$, setting $s:=(T^{-1}\overline{t})(x)$ we have $\overline{s}(x)=(T^{-1}\overline{t})(x)$, so, again by Lemma 7, $(T\overline{s})(x)=\overline{t}(x)=t$ i.e. $\phi (s)=t$. Thus, $\phi :\mathbb {R}\rightarrow \mathbb {R}$ is strictly increasing and surjective, hence continuous.
3. Define the function $\Phi$ on $X_{0}\times \mathbb {R}$ by:
  $$\begin{aligned} \Phi (x,s):=(T\overline{s})(x) \quad \quad (x\in X_{0}, s\in \mathbb {R}), \end{aligned}$$
  so that $F(x,s)=(x,\Phi (x,s))$ $(x\in X_{0},s\in \mathbb {R})$. We prove $\Phi$ to be continuous: Let $r\in \mathbb {R}$. We show that the set $A:=\{(x,s)\in X_{0}\times \mathbb {R}:\Phi (x,s)>r\}$ is open. Take $(x_{0},s_{0})\in A$; we make an open $W\subseteq X_{0}$ and an $s'\in \mathbb {R}$ for which:
  2 $$\begin{aligned} (x_{0},s_{0})\in W\times (s',\infty )\subseteq A. \end{aligned}$$
  We have $\Phi (x_{0},s_{0})>r$. By (2) there is an $s'<s_{0}$ with $\Phi (x_{0},s')>r$. The continuity of $T(\overline{s'})$ entails that the set $W:=\{x\in X_{0}:\Phi (x,s')>r\}$ is open in $X_{0}$. Of course it contains $x_{0}$. If $x\in W$ and $s\in (s',\infty )$, then $\Phi (x,s)\ge \Phi (x,s')>r$, so $(x,s)\in A$. We have Eq. (2). In the same way one shows that for every $r\in \mathbb {R}$ the set $\{(x,s)\in X_{0}\times \mathbb {R}:\Phi (x,s)<r\}$ is open. Thus, $\Phi$ is continuous, and so is *F*.
4. With the order automorphism $T^{-1}$ we associate a continuous $G:X_{0}\times \mathbb {R} \rightarrow X_{0}\times \mathbb {R}$:
  $$\begin{aligned} G(x,s)=(x,(T^{-1}\overline{s})(x)). \end{aligned}$$
  Then
  $$\begin{aligned} (x,(T^{-1}g)(x))=G(x,g(x)) \quad \quad (x\in X_{0},g\in C(X)). \end{aligned}$$
  In particular, for all (*x*, *s*) in $X_{0}\times \mathbb {R}$
  $$\begin{aligned} F(G(x,s))=F(x,(T^{-1}\overline{s})(x))=(x,(TT^{-1}\overline{s})(x))=(x,s), \end{aligned}$$
  and, similarly, $G(F(x,s))=(x,s)$. It follows that *F* is a homeomorphism $X_{0}\times \mathbb {R} \rightarrow X_{0}\times \mathbb {R}$. $\square$

Was the introduction of $X_{0}$ necessary? Or is $X_{0}$ an artefact of our reasoning and may we replace “$X_{0}$” by “*X*” in Eq. (1)? We consider these questions in the following paragraphs.

### Lemma 19

Let *X* be metrizable. Then $X_0=X$.

### Proof

Assume $x\in X\backslash X_{0}$, $s,t\in \mathbb {Q}$, $s<t$, $(T\overline{s})(x)=(T\overline{t})(x)$; it suffices to deduce a contradiction.

As $x\in X\backslash X_{0}$ and $X_{0}$ is dense there is a sequence $(x_{n})_{n\in \mathbb {N}}$ in $X_{0}$ that converges to *x* such that $x_{n}\not = x_{m}\not = x$ a soon as $n\not =m$. Let *Z* be the closed set $\{x,x_{1},x_{2},\ldots \}$. Define $g\in C(Z)$ by

$$\begin{aligned} \begin{aligned} g(x_{n})&= (T\overline{s})(x_{n}) \quad \text {if } \, n \, \text { is even},\\ g(x_{n})&= (T\overline{t})(x_{n}) \quad \text {if } \, n \, \text { is odd},\\ g(x)&= (T\overline{s})(x)=(T\overline{t})(x). \end{aligned} \end{aligned}$$

By Tietze’s Extension Theorem there is an *f* in *C*(*X*) such that $Tf=g$ on *Z*. From Lemma 7 we obtain $f(x_{n})=\overline{s}(x_{n})=s$ if *n* is even and $f(x_{n})=\overline{t}(x_{n})=t$ if *n* is odd. But then $f(x)=s$ and $f(x)=t$; contradiction. $\square$

### Example 20

An example to show that $X\backslash X_{0}$ may be non-empty.

For *X* we take the Stone–Čech compactification of $\mathbb {N}=\{1,2,3,\ldots \}$. We view $\mathbb {N}$ as a subset of *X*. There is an order (even: Riesz) isomorphism $l^{\infty }\rightarrow C(X)$ sending $c_{0}$ onto $\{f\in C(X):f=\overline{0} \text { on } X\backslash \mathbb {N}\}$.

For $n\in \mathbb {N}$ we make a strictly increasing bijection $\varphi _{n}:\mathbb {R}\rightarrow \mathbb {R}$ by

$$\begin{aligned} \varphi _{n}(t)={\left\{ \begin{array}{ll} t^n&{}\quad \text {if }\; 0\le t\le 1, \\ t &{}\quad \text {otherwise}.\end{array}\right. } \end{aligned}$$

The formula

$$\begin{aligned} (T_{0}a)_{n}:=\varphi _{n}(a_{n}) \quad \quad (a\in l^{\infty },n\in \mathbb {N}) \end{aligned}$$

defines an order isomorphism $T_{0}:l^{\infty }\rightarrow l^{\infty }$ with $T_{0}\overline{t}\in c_{0}$ for all $t\in [0,1)$. This $T_{0}$ determines an order isomorphism $T:C(X)\rightarrow C(X)$ with $T\overline{t}\in \{f\in C(X):f=\overline{0} \text { on } X\backslash \mathbb {N}\}$ for $t\in [0,1)$. For such *t* we get $T\overline{t}=T\overline{0}$ on $X\backslash \mathbb {N}$, so $X\backslash \mathbb {N} \subseteq X\backslash X_{0}$.

### Comment 21

The introduction of $X_{0}$ in Theorem 18 is not redundant. Indeed, suppose for every *X* and every vertically acting order isomorphism $T:C(X)\rightarrow C(X)$ there is an $F:X\times \mathbb {R} \rightarrow X\times \mathbb {R}$ such that for $f\in C(X)$,

$$\begin{aligned} (x,f(x))=F(x,(Tf)(x)) \quad \quad (x\in X). \end{aligned}$$

Now let *X*, *T* be as in Example 20. Take *x* in $X\backslash \mathbb {N}$ and $f=\overline{t}$ for some *t* in (0, 1). Then

$$\begin{aligned} (x,t)=F(x,(T\overline{t})(x))=F(x,0)=F(x,(T\overline{0})(x))=(x,0). \end{aligned}$$

Contradiction.

At last we want to prove what we have promised in the introduction.

### Theorem 22

Let *X*, *Y* be compact Hausdorff spaces, *T* an order isomorphism $C(X)\rightarrow C(Y)$, and $\sigma$ the associated homeomorphism $X\rightarrow Y$. Form $X_{0}$ as in Definition 6 and put $Y_{0}=\sigma (X_{0})$ ( so that $X\backslash X_{0}$ and $Y\backslash Y_{0}$ are meagre, and $X_{0}=X,Y_{0}=Y$ if *X* and *Y* are metrizable). Define $F:X_{0}\times \mathbb {R}\rightarrow Y_{0}\times \mathbb {R}$ by

$$\begin{aligned} F(x,s):=(\sigma (x),(T\overline{s})(\sigma (x))) \quad \quad (x\in X_{0},s\in \mathbb {R}). \end{aligned}$$

For $f\in C(X)$, set $\Gamma _{f}:=\{(x,f(x)):x \in X_{0}\}$. Similarly, make $\Gamma _{g}$ for $g\in C(Y)$.Then *F* is a homeomorphism and maps $\Gamma {f}$ onto $\Gamma _{Tf}$ $(f\in C(X))$.

### Proof

Define $\sigma ^{*}$ as in Definition 1 and $\sigma ^{*-1}$ as its inverse, set $S=(\sigma ^{*}\circ T)$ and note that $T=\sigma ^{*-1} \circ (\sigma ^{*}\circ T)=\sigma ^{*-1}\circ S$ and also that Lemma 14 proves that *S* is acting vertically. Define $B:X_{0}\times \mathbb {R}\rightarrow Y_{0}\times \mathbb {R}$ and $G:X_{0}\times \mathbb {R}\rightarrow X_{0}\times \mathbb {R}$ by

$$\begin{aligned} B(x,s)&:=(\sigma (x),s) \quad \quad (x\in X_{0},s\in \mathbb {R}), \\ G(x,s)&:=(x,(T\overline{s})(\sigma (x))) \quad \quad (x\in X_{0},s\in \mathbb {R}). \end{aligned}$$

First of all note that $F=B\circ G$. Secondly note that since $\sigma$ is a homeomorphism we know that *B* is continuous. Thirdly we see that $G(x,(T\overline{s})(\sigma (x)))=G(x,(S\overline{s})(x))$ which enables us to fall back on Theorem 18 for the proof that *G* is continuous and that *G* maps $\Gamma _{f}$ onto $\Gamma _{Sf}$ ($f\in C(X)$). We conclude that $F=B\circ G$ is continuous and maps $\Gamma _{f}$ onto $B\circ \Gamma _{Sf}=\Gamma _{\sigma ^{*-1}\circ Sf}=\Gamma _{Tf}$ ($f\in C(X)$). $\square$

Theorem 22 is an amplification of known representation theorems such as Theorem 1(*b*) in [5]. This paper gives further representations of lattice homomorphisms $C(X)\rightarrow C(Y)$ that are not required to be isomorphisms, and for isomorphisms that are norm-continuous. It also considers order isomorphisms $L^{\infty }(\mu )\rightarrow L^{\infty }(\nu )$ where $\mu$ and $\nu$ are measures.

A related paper is [6], dealing with order isomorphisms between sufficiently large subspaces of *C*(*X*) and *C*(*Y*) for metrizable *X* and *Y*.

## Concerning $C^{\infty }(X)$

$\overline{\mathbb {R}}$ is the extended real number system $\mathbb {R}\cup \{\infty \}\cup \{-\infty \}$.

### Definition 23

Let *Z* be a compact Hausdorff space.

1. A subset of *Z* is called *clopen* if it is both closed and open. *Z* is *extremally disconnected* if the closure of every open set is open (hence, clopen). This is the case if and only if the Riesz space *C*(*Z*) is Dedekind complete. If *Z* is extremally disconnected, the formula
  $$\begin{aligned} U\leftrightarrow B_{U} \end{aligned}$$
  (See Notation 9 for $B_{U}$) establishes a one-to-one correspondence between the clopen subsets of *Z* and the bands of *C*(*Z*).
2. Let *Z* be, indeed, extremally disconnected. By $C_{\overline{\mathbb {R}}}(Z)$ we denote the collection of all continuous functions $Z\rightarrow \overline{\mathbb {R}}$. As an ordered set, $C_{\overline{\mathbb {R}}}(Z)$ is isomorphic to
  $$\begin{aligned} \{h\in C(Z):-\mathbbm {1} \le h\le \mathbbm {1}\}. \end{aligned}$$
  It follows that $C_{\overline{\mathbb {R}}}(Z)$ is a complete lattice and is completely distributive (i.e., if $h_{0}=\sup H$ in $C_{\overline{\mathbb {R}}}(Z)$, then $h_{0}\wedge h_{1}=\sup \{h\wedge h_{1}:h\in H\}$ for all $h\in C_{\overline{\mathbb {R}}}(Z)$).
3. Again, let *Z* be extremally disconnected. By
  $$\begin{aligned} C^{\infty }(Z) \end{aligned}$$
  we denote the set of all continuous $f:Z\rightarrow \overline{\mathbb {R}}$ for which the closed set $\{z:|f(z)|=\infty \}$ has empty interior. For $f,g\in C^{\infty }(Z)$ there is a unique $f+g$ in $C^{\infty }(Z)$ with the property that
  $$\begin{aligned} (f+g)(z)=f(z)+g(z)\quad \text { if }\quad |f(z)|,|g(z)|<\infty . \end{aligned}$$
  Under the addition “$+$”, $C^{\infty }(Z)$ is a Riesz space that contains *C*(*Z*) as an order dense Riesz ideal. For details we refer to page 189 of [1].

### Theorem 24

Let *X* and *Y* be extremally disconnected compact Hausdorff spaces and let *T* be an order isomorphism $C^{\infty }(X)\rightarrow C^{\infty }(Y)$. Then *X* and *Y* are homeomorphic. There exists a (unique) homeomorphism $\sigma :X\rightarrow Y$ satisfying:

$$\begin{aligned} x\in X,f,g\in C^{\infty }(X),f(x)<g(x)\quad \Rightarrow \quad (Tf)(\sigma x)\le (Tg)(\sigma x) \end{aligned}$$

### Proof

Copy the proof of Theorem 2, just replacing “*C*” by “$C^{\infty }$” and, in (2), inserting the condition $g(a),h(a)\not = -\infty$. This does not affect the rest of the proof. $\square$

### Comment 25

For later use we file away this obvious consequence: Let *X* and *Y* be extremally disconnected compact Hausdorff spaces. If $C^{\infty }(X)$ and $C^{\infty }(Y)$ are order isomorphic, then they are Riesz isomorphic.

### Definition 26

We define “associated” as in Definition 4, “acting horizontally” and “acting vertically” as in Definition 12.

The natural analogues of Theorem 11 and Lemma 14 and Theorem 17 are valid.

### Definition 27

We define the set $X_{0}$ verbatim as in Definition 6.

One extra observation: If $x\in X_{0}$ and $f\in C^{\infty }(X)$, then

$$\begin{aligned} f(x)=\infty \quad \Leftrightarrow \quad (Tf)(\sigma x)=\infty \end{aligned}$$

(and $f(x)=-\infty \Leftrightarrow (Tf)(x)=-\infty$). For a proof, suppose $f(x)<\infty$; we show that $(Tf)(\sigma x)<\infty$. There exist $s,t\in \mathbb {Q}$ with $f(x)<s<t$. Then $(Tf)(\sigma x) \le (T\overline{s})(\sigma x)<(T\overline{t})(\sigma x)$, so $(Tf)(\sigma x)<\infty$.

### Theorem 28

Let *X* be extremally disconnected and let *T* be a vertically acting order isomorphism $C^{\infty }(X)\rightarrow C^{\infty }(X)$. Take $X_{0}$ as in Definition 27. Define $F:X_{0}\times \overline{\mathbb {R}}\rightarrow X_{0}\times \overline{\mathbb {R}}$ byThen *F* is a homeomorphism and for every $f\in C^{\infty }(X)$ we have

3 $$\begin{aligned} (x,(Tf)(x))=F(x,f(x)) \quad \quad (x\in X_{0}). \end{aligned}$$

### Proof

(Essentially rephrasing the proof of Theorem 18)

1. Equation (3) follows from Lemma 7 and the observation in Definition 27.
2. Take $x\in X_{0}$ and consider the function $\overline{\phi }:\overline{\mathbb {R}}\rightarrow \overline{\mathbb {R}}$ defined by:
  $$\begin{aligned} \begin{aligned} \overline{\phi }(s):=(T\overline{s})(x) \quad \quad (s\in \mathbb {R}) \\ \overline{\phi }(\infty ):=\infty ,\overline{\phi }(-\infty ):=-\infty \end{aligned} \end{aligned}$$
  From (2) of the proof of Theorem 18 one sees that the restriction of $\overline{\phi }$ is a strictly increasing bijection $\mathbb {R}\rightarrow \mathbb {R}$. Consequently $\overline{\phi }$ itself is a strictly increasing bijection $\overline{\mathbb {R}}\rightarrow \overline{\mathbb {R}}$, hence is continuous.
3. From here onwards, follow the proof of Theorem 18, replacing $\Phi$ by $\overline{\Phi }:X_{0}\times \overline{\mathbb {R}}\rightarrow \overline{\mathbb {R}}$ defined by: $\square$

## Arbitrary Archimedean Riesz spaces

### Definition 29

1. Following [1] we define an Archimedean Riesz space to be *universally complete* if it is both Dedekind and laterally complete. This is the case if and only if it is Riesz isomorphic to $C^{\infty }(Z)$ for some extremally disconnected compact Hausdorff space *Z*.
2. Let *E* be an Archimedean Riesz space. There exists a universally complete Riesz space *F* such that *E* is Riesz isomorphic to some order dense Riesz subspace of *F*. Such an *F* is called a *universal completion* of *E*. All universal completions of *E* are Riesz isomorphic.

In this section we consider an order isomorphism *T* between two Riesz spaces, *E* and *F*. We show that there exists an extremally disconnected compact Hausdorff space *X* such that $C^{\infty }(X)$ is a universal completion of *E* and of *F*, and such that, if we identify *E* and *F* with suitable Riesz subspaces of $C^{\infty }(X)$, the map *T* extends uniquely to a vertically acting order isomorphism $C^{\infty }(X)\rightarrow C^{\infty }(X)$.

### Observations 30

The following is preparation for the proof of Lemma 32. Here *E* and *F* are Riesz spaces, *T* is an order isomorphism $E\rightarrow F$ with $T0=0$.

1. The formula
  $$\begin{aligned} T^*f:=-T(-f) \quad \quad (f\in E) \end{aligned}$$
  defines an order isomorphism $T^*:E\rightarrow F$ with $T^*0=0$.
2. For $f\in E$ we have
  $$\begin{aligned} (Tf)^+=Tf^+, (Tf)^-=T^*f^-. \end{aligned}$$
  Indeed, $$(Tf)^+=Tf\vee T0=T(f\vee 0)=Tf^+$$, $$(Tf)^-=(-Tf)^+=(T^*(-f))^+=T^*((-f)^+)=T^*f^-$$.
3. Hence,
  $$\begin{aligned} Tf=Tf^+-T^*f^- \quad \quad (f\in E). \end{aligned}$$
4. Let $$f,g\in E$$. Then
  $$\begin{aligned} f\perp g \quad \Leftrightarrow \quad Tf\perp Tg. \end{aligned}$$
  For a proof, assume $f\perp g$. Then $(f+g)^+=f^+\vee g^+$ and $(f+g)^-=f^-\vee g^-$. By (2)
  $$\begin{aligned} \begin{aligned} (T(f+g))^+&=T((f+g)^+)=T(f^+\vee g^+)=Tf^+\vee Tg^+ \\ (T(f+g))^-&=T^*((f+g)^-)=T^*(f^-\vee g^-)=T^*f^- \vee T^*g^-. \end{aligned} \end{aligned}$$
  Hence, the elements $Tf^+,Tg^+,T^*f^-,T^*g^-$ of $F^+$ are pairwise disjoint. Then $Tf^+-T^*f^-\perp Tg^+-T^*g^-$, i.e., $Tf\perp Tg$.

As a consequence of (4) we obtain.

### Theorem 31

Let *E* and *F* be Archimedean Riesz spaces and let $T:E\rightarrow F$ be an order isomorphism, $T0=0$. Then *T* maps every band of *E* onto a band of *F*.

### Proof

(We apply [3], 19.2 and 22.3) Let *B* be a band in *E*. There is a subset *A* of *E* with $B:=\{f\in E:f\perp h \text { for all } h\in A\}$. Then $T(B)=\{g\in F:g\perp j \text { for all } j\in T(A)\}$, so *T*(*B*) is a band in *F*. $\square$

### Lemma 32

Let *X* and *Y* be extremally disconnected compact Hausdorff spaces. Let $E\subseteq C^{\infty }(X)$ and $F\subseteq C^{\infty }(Y)$ be order dense Riesz subspaces and $T:E\rightarrow F$ an order isomorphism. Then *T* extends uniquely to an order isomorphism $\overline{T}:C^{\infty }(X)\rightarrow C^{\infty }(Y)$.

### Proof

1. The map $S:f\rightarrow Tf-T\overline{0}$ is an order isomorphism $E\rightarrow F$ sending $\overline{0}$ to $\overline{0}$, and if *S* has a unique extension, so has *T*. Thus, we may (and do) assume $T\overline{0}=\overline{0}$.
2. Let $R_{1}$ and $R_{2}$ be order isomorphisms $C^{\infty }(X)\rightarrow C^{\infty }(Y)$ that extend *T*; we prove $R_{1}=R_{2}$. Thanks to order denseness, for $h\in C^{\infty }(X)^+$ we have
  $$\begin{aligned} R_{1}h=\sup _{\begin{array}{c} f\in E^+ \\ f\le h \end{array}}R_{1}f=\sup _{\begin{array}{c} f\in E^+ \\ f\le h \end{array}}Tf=R_{2}h. \end{aligned}$$
  Using the notations of Observations 30, we similarly obtain $R^*_{1}=R^*_{2}$ on $C^{\infty }(X)^+$, and thereby
  $$\begin{aligned} R_{1}h=R_{1}h^+-R^*_{1}h^-=R_{2}h^+-R_{2}^*h^-=R_{2}h\quad \quad (h\in C^{\infty }(X)). \end{aligned}$$
3. Let $h\in C^{\infty }(X)^+$. In $C_{\overline{\mathbb {R}}}(Y)$ (see Definition 23 (2)) the set $\{Tf:f\in E^+,f \le h\}$ has a supremum, $\overline{h}$. Claim: for $g\in E^+$ we have:
  $$\begin{aligned} g\le h \Leftrightarrow Tg\le \overline{h}. \end{aligned}$$
  Indeed, the implication “$\Rightarrow$” is clear from the definition of $\overline{h}$. For the reverse implication, assume $Tg\le \overline{h}$. Then in $C_{\overline{\mathbb {R}}}(Y)$:
  $$\begin{aligned} Tg=Tg\wedge \overline{h}=Tg\wedge \sup _{\begin{array}{c} f\in E^+ \\ f\le h \end{array}}Tf=\sup _{\begin{array}{c} f\in E^+ \\ f\le h \end{array}}T(g\wedge f). \end{aligned}$$
  Hence, by order denseness, $Tg=\sup \{T(g\wedge f):f\in E^+,f\le h\}$ in *F*, implying $g=\sup \{g\wedge f:f\in E^+,f\le h\}$ in *E*, and $g\le h$.
4. Take $h,\overline{h}$ as above. We prove $\overline{h}\in C^{\infty }(Y)^+$. To this end, it suffices to show that the closed set $\{y:\overline{h}(y)=\infty \}$ has empty interior. Let *U* be the (clopen) interior of $\{y:\overline{h}(y)=\infty \}$. As $T^{-1}$ maps bands of *F* to bands of *E*, the set $B:=\{g\in E:Tg=\overline{0} \text { on } Y\backslash U\}$ is a band in *E*. But $B^+\subseteq \{g\in E^+:Tg\le \overline{h}\}=\{g\in E^+:g\le h\}$ by (3). Hence, $B=\{0\}$, $U=\emptyset$, and $\overline{h}\in C^{\infty }(Y)^+$.
5. From (3) and (4) we see that the formula
  $$\begin{aligned} Ah=\sup \{Tf:f\in E^+,f\le h\} \end{aligned}$$
  defines an (increasing) map $A:C^{\infty }(X)^+\rightarrow C^{\infty }(Y)^+$ for which
  $$\begin{aligned} Tg\le Ah \Leftrightarrow g\le h \quad \quad (h\in C^{\infty }(X)^+,g\in E^+). \end{aligned}$$
  Note that $Ah=Th$ if $h\in E^+$. In the same way, the order isomorphism $T^{-1}:F\rightarrow E$ generates an increasing $A':C^{\infty }(Y)^+\rightarrow C^{\infty }(X)^+$ with
  $$\begin{aligned} T^{-1}f\le A'j\Leftrightarrow f\le j \quad \quad (j\in C^{\infty }(Y), f\in F^+), \end{aligned}$$
  which is the same as
  $$\begin{aligned} g\le A'j\Leftrightarrow Tg\le j \quad \quad (j\in C^{\infty }(Y), g\in E^+). \end{aligned}$$
  For $h\in C^{\infty }(X)^+$ we obtain
  $$\begin{aligned} g\le A'Ah\Leftrightarrow Tg\le Ah \Leftrightarrow g\le h \quad \quad (g\in E^+), \end{aligned}$$
  so $A'Ah=h$ by the order denseness of *E*. Thus, $A'A$ is the identity map of $C^{\infty }(X)^+$. By symmetry, $AA'$ is the identity map on $C^{\infty }(Y)^+$: The maps *A* and $A'$ are order isomorphisms and each other’s inverses.
6. Just as *T* engenders *A*, so $T^*$ (see Observations 30) leads to an order isomorphism $B:C^{\infty }(X)^+\rightarrow C^{\infty }(Y)^+$ satisfying $Bh=T^*h$ for $h\in E^+$. Led by Observations 30, we define $\overline{T}:C^{\infty }(X)\rightarrow C^{\infty }(Y)$ by
  $$\begin{aligned} \overline{T}h:=Ah^+-Bh^- \quad \quad (h\in C^{\infty }(X)). \end{aligned}$$
  Observe that $\overline{T}$ is an extension of *T*. We conclude our proof by showing that $\overline{T}$ is an order isomorphism. Firstly, $\overline{T}$ is increasing: if $h\le j$ in $C^{\infty }(X)$, then $h^+\le j^+$ and $h^-\ge j^-$, whence $\overline{T}h\le \overline{T}j$. Secondly, for all $h\in C^{\infty }(X)$, $Ah^+$ lies in the band generated by $h^+$ (look at the definition) and, similarly $Bh^-$ lies in the band generated by $h^-$. Hence $Ah^+\perp Bh^-$, and
  $$\begin{aligned} (\overline{T}h)^+=Ah^+, (\overline{T}h)^-=Bh^-. \end{aligned}$$
  Thirdly, the formula $Sj:=A^{-1}j^+-B^{-1}j^-$ defines a map $S:C^{\infty }(Y)\rightarrow C^{\infty }(X)$ with properties similar to those of $\overline{T}$. In particular
  $$\begin{aligned} (Sj)^+=A^{-1}j^+,(Sj)^-=B^{-1}j^- \quad (j\in C^{\infty }(Y)). \end{aligned}$$
  Then for all $h\in C^{\infty }(X)$
  $$\begin{aligned} S\overline{T}h=A^{-1}((\overline{T}h)^+)-B^{-1}((\overline{T}h)^-)=A^{-1}Ah^+-B^{-1}Bh^-=h \end{aligned}$$
  and $S\overline{T}$ is the identity map of $C^{\infty }(X)$. Also, $\overline{T}S$ is the identity map of $C^{\infty }(Y)$. It follows that $\overline{T}$ and *S* are order isomorphisms. $\square$

In terms of abstract Riesz space theory, the lemma says:

### Theorem 33

An order isomorphism between two Archimedean Riesz spaces extends uniquely to an order isomorphism between their universal completions.

### Corollary 34

Let *E* be a universally complete Riesz space. If a Riesz space *F* is order isomorphic to *E*, then *F* is even Riesz isomorphic to *E*.

### Proof

As *E* is Dedekind complete, so is *F*. In particular, *F* is Archimedean. Let $F^u$ be a universal completion of *F*. Choose an order isomorphism $T:E\rightarrow F$. By Theorem 33, this *T* “extends” to an order isomorphism $E\rightarrow F^u$. Then $F^u=F$ and *F* is universally complete. Now apply Definition 29(1) and Theorem 24. $\square$

### Theorem 35

Let *E* and *F* be Archimedean Riesz spaces and *T* an order isomorphism $E\rightarrow F$. Then there exist an extremally disconnected compact Hausdorff space *X*, order dense Riesz subspaces $\hat{E}$ and $\hat{F}$ of $C^{\infty }(X)$, Riesz isomorphisms $R_{E}:E\rightarrow \hat{E}$ and $R_{F}:F\rightarrow \hat{F}$, and a vertically acting order isomorphism $\hat{T}:C^{\infty }(X)\rightarrow C^{\infty }(X)$ such that $R_{F}T=\hat{T}R_{E}$:


### Proof

Choose extremally disconnected compact Hausdorff spaces *X* and *Y* such that $C^{\infty }(X)$ and $C^{\infty }(Y)$ are universal completions of *E* and *F*, respectively. Choose a Riesz isomorphism $R_{E}$ of *E* onto an order dense Riesz subspace $\hat{E}$ of $C^{\infty }(X)$, and a Riesz isomorphism *A* of *F* onto an order dense Riesz subspace *A*(*F*) of $C^{\infty }(Y)$.

We have an order isomorphism $S:\hat{E}\rightarrow A(F)$ given by $SR_{E}=AT$. By Lemma 32 this *S* extends to an order isomorphism $\overline{S}:C^{\infty }(X)\rightarrow C^{\infty }(Y)$. Let $\sigma :X\rightarrow Y$ be the homeomorphism associated with $\overline{S}$. Then $\sigma$ induces a Riesz isomorphism $\sigma ^*:C^{\infty }(Y)\rightarrow C^{\infty }(X)$.

Put $\hat{F}:=\sigma ^*(A(F))$, $R_{F}:=\sigma ^*A$, and $\hat{T}:=\sigma ^*\overline{S}$. Now $\hat{F}$ is an order dense Riesz subspace of $C^{\infty }(X)$, $R_{F}$ is a Riesz isomorphism $F\rightarrow \hat{F}$, $\hat{T}$ is an order isomorphism $C^{\infty }(X)\rightarrow C^{\infty }(X)$ that acts vertically (Lemma 14, extended) and $R_{F}T=\sigma ^*AT=\sigma ^*SR_{E}=\hat{T}R_{E}$. $\square$

We are grateful to the referee for valuable comments.
